# Sulfur-Rich Garlic Extract (DNR) as a Promising Natural Therapeutic for Diabetic Nephropathy: Evidence from a *db*/*db* Mouse Model

**DOI:** 10.3390/ijms262010184

**Published:** 2025-10-20

**Authors:** Ju Hee Park, Byung Sik Cho, Xue Bi Zhou, Richard Kyung, Myong Jo Kim

**Affiliations:** 1 Department of Applied Plant Sciences, Kangwon National University, Chuncheon 24341, Republic of Korea; sj2024@kangwon.ac.kr (J.H.P.);; 2 K-Future Clinic, Gwangju 12816, Republic of Korea; heartcho21@hanmail.net (B.S.C.);

**Keywords:** sulfur-rich garlic extract (DNR), amelioration of diabetic nephropathy, *db*/*db* mouse model, anti-fibrosis, KIM-1/TGF-β1

## Abstract

Diabetic nephropathy (DNR) remains a major complication of type 2 diabetes with limited options to halt progression. We evaluated whether DNR (a sulfur-rich extract from Hongsan garlic) confers renoprotection in a *db*/*db* mouse model. Seventy male C57BLKS/J mice were randomized into seven groups (*db*/*m* control, db/*db* control, metformin 250 mg/kg, DNR 100/300/900 mg/kg, and metformin 250 mg/kg + DNR 300 mg/kg) and treated orally for eight weeks. Physiological, biochemical, urinary, histological, and immunohistochemical(IHC) endpoints were assessed, including serum creatinine, blood urea nitrogen(BUN), lipids, glucose, urinary microalbumin/albumin-to-creatinine ratio(ACR), glomerular area, mesangial expansion, and renal KIM-1 and TGF-β1 expression. Chemical profiling of the DNR extract by HPLC and LC–MS/MS identified allicin as a principal sulfur-containing constituent, exhibiting a distinct retention peak at 2.90 min and a protonated molecular ion at *m*/*z* 162.1 [M]^+^ with diagnostic fragment ions at *m*/*z* 145.1, 120.1, and 99.0. Allicin was qualitatively confirmed as a characteristic component of DNR, serving as a representative chemical marker for compositional characterization. DNR produced dose-dependent improvements: reductions in serum creatinine and BUN, improved lipid and glycemic profiles, decreased urinary microalbumin and ACR, and amelioration of glomerular hypertrophy and mesangial matrix expansion. IHC showed lower KIM-1 and TGF-β1 staining in treated groups. Effects at higher DNR doses were comparable to or additive with metformin for several endpoints. These findings indicate that DNR has promising renoprotective effects in this preclinical model.

## 1. Introduction

Type 2 diabetes (T2D) is a leading cause of diabetic kidney disease (DKD) worldwide and is a well-established risk factor for renal failure and cardiovascular disease. At the cellular level, hyperglycemia triggers dysregulated intracellular pathways, excessive extracellular matrix accumulation, and glomerular basement membrane (GBM) thickening. Persistent inflammation, mediated by increased cytokine and chemokine production, drives fibrosis and disrupts vascular integrity. These processes collectively contribute to the development and progression of diabetic nephropathy by intensifying inflammation, fibrosis, endothelial dysfunction, and podocyte injury. Comprehensive investigation of these pathological mechanisms is essential for formulating advanced therapeutic approaches [1].

Current traditional management strategies for DKD primarily focus on glycemic control and blood pressure management, which are somewhat effective in reducing albuminuria; however, they show limited efficacy in preventing the decline of glomerular filtration rate (GFR) or the progression to end-stage renal disease (ESKD) [2].

To address these shortcomings, new pharmacologic interventions such as SGLT-2 inhibitors, GLP-1 receptor agonists, and non-steroidal mineralocorticoid receptor antagonists have been introduced, demonstrating significant potential for renal protection and cardiovascular benefit. These drugs are anticipated to decelerate the progression of DKD and enhance the quality of life for patients [2]. In addition to synthetic drug development, investigations involving natural products are increasingly recognized for their capacity to improve kidney disease outcomes. Due to their low side effect profiles and high biocompatibility, a safe and effective long-term treatment alternative, highlighting the necessity for more rigorous research in this domain. According to Zhou et al. [3], natural compounds may counteract renal fibrosis by mediating anti-inflammatory and antioxidant mechanisms, which safeguard kidney structure and function. This underscores the potential of natural materials as a significant alternative in kidney disease management.

Garlic is widely recognized for its diverse medicinal and therapeutic effects, primarily attributed to its sulfur-containing constituents such as allicin, ajoene, and vinyl-dithiin. Upon crushing garlic, allicin rapidly converts to derivatives including diallyl sulfide (DAS), diallyl disulfide (DADS), diallyl trisulfide (DATS), and ajoene. Notably, derivatives like DATS are sources of hydrogen sulfide (H_2_S), which contributes to key physiological processes such as vasodilation, thereby potentiating the health-promoting effects of garlic [4]. As an endogenous gasotransmitter, H_2_S is essential for enhancing vascular function and blood circulation, promoting vasodilation, improving blood flow, and mitigating oxidative stress [5].

This is particularly important in renal health, as glomerular fibrosis is a major contributor to chronic kidney disease (CKD) and is associated with progressive loss of kidney function. Evidence indicates that antifibrotic treatments can reduce renal fibrosis and decelerate the decline in estimated glomerular filtration rate (eGFR). Both sulfur and allicin demonstrate antifibrotic effects, which have been documented in several kidney disease models, such as obstructive nephropathy, diabetic nephropathy, and aging-related renal injury. These effects operate through mechanisms including inhibition of the TGF-β/SMAD signaling pathways, reduction in oxidative stress, and modulation of autophagy and inflammatory pathways. Notably, allicin counteracts fibrosis progression by suppressing the TGF-β signaling cascade, diminishing oxidative stress and inflammatory responses, and regulating apoptosis [6,7].

In addition, both sulfur and allicin have demonstrated chelating activity, as illustrated by numerous studies examining the role of sulfur and sulfur-containing molecules in metal detoxification. Allicin has been shown to chelate heavy metals, specifically such as lead (Pb^2+^), copper (Cu^2+^), and cadmium (Cd^2+^). These studies suggest that allicin serves both as a natural chelator and an antioxidant, a function attributed to its organosulfur configuration and the presence of thiol (-SH) or sulfoxide groups [8,9].

DNR is derived from Hongsan garlic and contains a high concentration of allicin, utilizing mineral water enriched with organic sulfur and sulfate to potentiate the activity of these sulfur compounds. DNR’s chelation therapy not only aids in the elimination of heavy metals but also promotes the dispersion of aggregated red blood cells, thereby restoring typical cellular morphology. Through this process, there is a concomitant decrease in the blood levels of fats, lipoproteins, abnormal globulins, and fibrinogen, as seen through reduced erythrocyte aggregation. As the protein-fibrin conglomerates dissipate, blood flow is improved, which in turn supports enhanced estimated glomerular filtration rate (eGFR). In this mechanism, sulfate (SO_4_^2−^) is essential for both detoxification and anti-inflammatory activity. By harnessing sulfate’s properties in these processes, DNR provides an evidence-based strategy for the management of diabetic nephropathy.

Building upon the established pharmacological and therapeutic attributes of garlic, this research investigates the renoprotective capabilities of high-sulfur garlic extracts (DNR). The study examines the potential of these high-sulfur formulations to alleviate diabetic nephropathy, emphasizing their role in renal defense and the promotion of favorable health outcomes. By reinforcing the medicinal promise of high-sulfur garlic extracts, this work supports the advancement of natural, biocompatible therapeutic options for the management of diabetic kidney disease.

## 2. Results

### 2.1. HPLC and LC–MS/MS Results of Allicin (Results)

**Figure 1:**
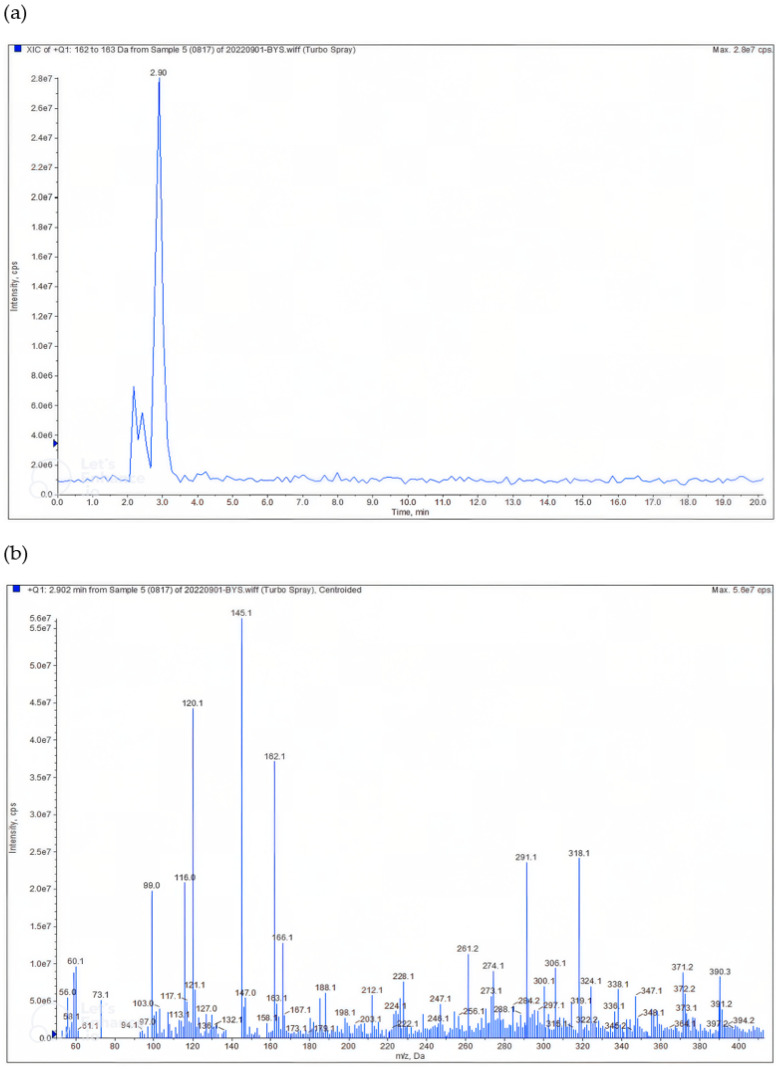
HPLC and LC–MS/MS analysis of allicin in Hongsan garlic extract. (**a**) Representative HPLC chromatogram of Hongsan garlic extract showing a distinct peak corresponding to allicin at a retention time of 2.90 min. (**b**) LC–MS/MS spectrum obtained at 2.90 min, exhibiting the protonated molecular ion at *m*/*z* 162.1 [M]^+^ along with characteristic fragment ions at *m*/*z* 145.1, 120.1, and 99.0, confirming the identity of allicin.

The HPLC chromatogram of the Hongsan garlic extract showed a distinct peak corresponding to allicin at a retention time of 2.90 min (Figure 1a). The sharp and symmetrical peak confirmed the selective detection of allicin under the applied conditions. No significant interfering peaks were observed, demonstrating the specificity of the method.

The LC–MS/MS spectrum obtained at 2.90 min further validated the identity of allicin (Figure 1b). The mass spectrum revealed the expected protonated molecular ion at *m*/*z* 162.1 [M]^+^, along with characteristic fragment ions at *m*/*z* 145.1, 120.1, and 99.0, consistent with the known fragmentation pattern of allicin. These results confirmed that allicin was present as a principal constituent of the garlic extract and can be reliably used as a marker compound for quality control of DNR.

### 2.2. Mortality Rate, Body Weight Changes, and Kidney Weight

**Figure 2:**
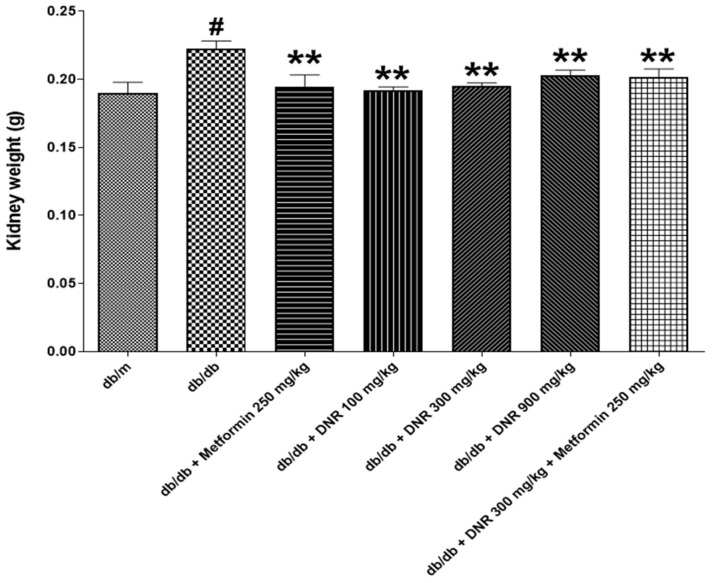
Effects of DNR treatment on kidney weight in *db*/*db* mice. #: Significant difference compared with the *db*/*m* (Student’s *t*-test), *p* < 0.05. **: Significant difference compared with the *db*/*db* (one-way ANOVA), *p* < 0.01.

Throughout the experimental period, neither mortality nor unusual clinical symptoms occurred in any treatment group, and no statistically significant body weight changes were detected; consequently, these findings were omitted from the manuscript. However, as shown in Figure 2, kidney weight measurements demonstrated a statistically significant increase in the *db*/*db* group (*p* < 0.01), consistent with the established pathophysiological features of diabetic nephropathy. This disease is commonly characterized by kidney hypertrophy due to hyperglycemia and associated metabolic dysfunction, as reported by Kowluru et al. [10].

### 2.3. Effects of DNR on Lipid Levels in db/db Mic

In this investigation, C57BLKs/J mice carrying an autosomal recessive mutation on chromosome 4 affecting the *db* gene, which encodes the leptin receptor, were utilized. This genetic alteration causes spontaneous hyperinsulinemia in these animals. Consequently, the mice develop obesity, hyperglycemia, hyperinsulinemia, and insulin resistance within the first month after birth. The co-occurrence of these metabolic abnormalities fosters the onset of diabetic nephropathy, establishing this model as particularly suitable for related research [11].

**Figure 3:**
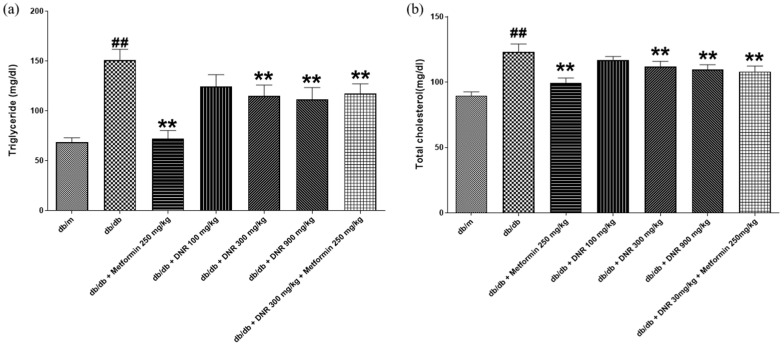
Effects of DNR treatment on metabolic parameters in *db*/*db* mice. (**a**) Triglyceride levels; (**b**) total cholesterol levels. ##: Significant difference compared with the *db*/*m* (Student’s *t*-test), *p* < 0.01. **: Significant difference compared with the *db*/*db* (one-way ANOVA), *p* < 0.01.

Analysis of triglyceride levels, as displayed in Figure 3a, showed a statistically significant elevation (*p* < 0.01) in the *db*/*db* mouse model. Administration of DNR at both 300 and 900 mg/kg, as well as MET 250 + DNR 300 mg/kg and MET 250 mg/kg, resulted in significant decreases in triglyceride concentrations in the experimental groups (*p* < 0.01). Likewise, total cholesterol analysis in Figure 3b indicated a substantial rise (*p* < 0.01) in the *db*/*db* mouse model. Treatment with DNR at both 300 and 900 mg/kg, alongside MET at 250 mg/kg + DNR at 300 mg/kg, yielded significant reductions in total cholesterol in animals administered MET at 250 mg/kg (*p* < 0.01).

### 2.4. Modulation of Serum and Urinary Glucose Levels by DNR in db/db Mice

Urinary glucose measurements can serve as indicators of renal functional status. In diabetic conditions, persistent hyperglycemia frequently causes increased urinary glucose elimination. Consequently, monitoring urinary glucose provides valuable insight into renal glucose handling capacity and helps evaluate the effects of pharmaceutical interventions on kidney function [12].

**Figure 4:**
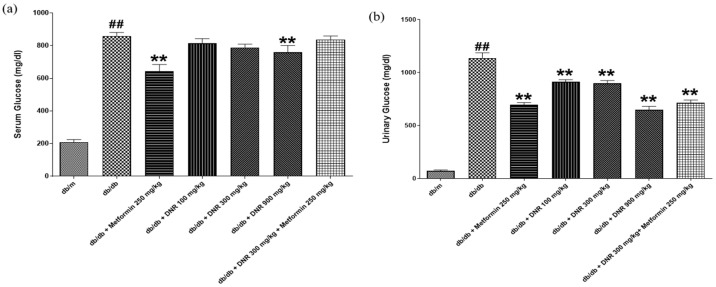
Effects of DNR treatment on glucose levels in *db*/*db* mice. (**a**) Serum glucose levels; (**b**) urinary glucose levels. ##: Significant difference compared with the *db*/*m* (Student’s *t*-test), *p* < 0.01. **: Significant difference compared with the *db*/*db* (one-way ANOVA), *p* < 0.01.

Analysis of serum and urinary glucose concentrations, as depicted in Figure 4a,b, demonstrates significant influences from both DNR and the positive control, Metformin (MET), on glucose regulation in the *db*/*db* mouse model. In Figure 4a, serum glucose concentrations in *db*/*db* mice were markedly higher than those in the *db*/m control group. Administration of Metformin (250 mg/kg) and different DNR doses (100 mg/kg, 300 mg/kg, and 900 mg/kg) led to a substantial decline (*p* < 0.01) in serum glucose concentrations, confirming that both DNR and MET effectively lower hyperglycemia in diabetic mice.

Figure 4b illustrates a comparable pattern in urinary glucose concentrations, with *db*/*db* mice displaying markedly elevated glucose levels relative to the *db*/*m* control group. Administration of MET and varying doses of DNR resulted in a significant reduction in urinary glucose (*p* < 0.01), indicating that these treatments not only lowered serum glucose but also diminished urinary glucose excretion, thereby reflecting enhanced glycemic control. Collectively, these results underscore the potential utility of DNR as an effective investigational compound, with MET serving as a positive control, for modulating glucose concentrations in both serum and urine.

### 2.5. Renal Protective Effects of DNR on Serum Creatinine and Blood Urea Nitrogen Levels in db/db Mice

**Figure 5:**
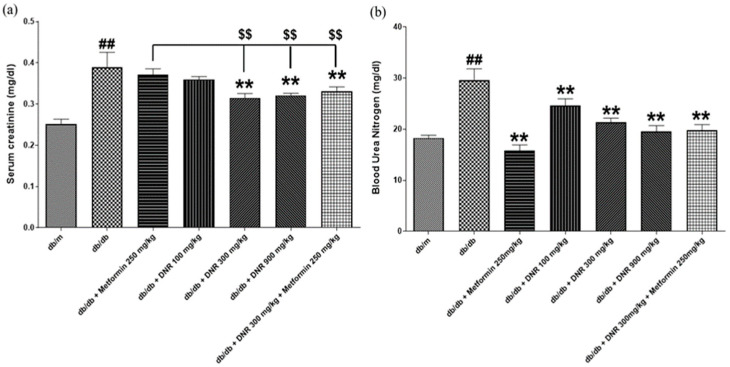
Effects of DNR treatment on renal function markers in *db*/*db* mice. (**a**) Serum creatinine levels; (**b**) blood urea nitrogen levels. ##: Significant difference compared with the *db*/*m* (Student’s *t*-test), *p* < 0.01. **: Significant difference compared with the *db*/*db* (one-way ANOVA), *p* < 0.01. $$: Significant difference compared with the *db*/*db* + Metfomin (one-way ANOVA), *p* < 0.01.

As illustrated in Figure 5a, the analysis of serum creatinine levels demonstrated a significant elevation in the *db*/*db* group compared to the *db*/*m* group (*p* < 0.01). Administration of Metformin at a dose of 250 mg/kg failed to significantly alter serum creatinine levels. In contrast, DNR treatment at both 300 mg/kg and 900 mg/kg, as well as the combined DNR 300 mg/kg with Metformin group, produced a significant decrease in serum creatinine levels (*p* < 0.01). These findings indicate a potential renoprotective effect of DNR in the diabetic state.

Figure 5b displays the evaluation of blood urea nitrogen (BUN) levels, revealing that the *db*/*db* group exhibited markedly elevated BUN levels relative to the *db*/*m* group (*p* < 0.01). All treatment groups, including Metformin and the respective doses of DNR, significantly reduced BUN levels (*p* < 0.01).

The *db*/*db* group showed a pronounced increase in serum creatinine levels compared to the *db*/*m* control group, consistent with renal impairment typically observed in diabetes. While Metformin alone failed to alter serum creatinine levels, DNR administered at doses of 300 mg/kg and 900 mg/kg significantly lowered these levels. Additionally, concurrent administration of DNR and Metformin produced a significant reduction in serum creatinine, suggesting that DNR may play a key role in alleviating diabetic renal injury.

### 2.6. DNR Improved Urinary Creatinine, Microalbumin, and ACR by DNR in db/db Diabetic Mice

**Figure 6:**
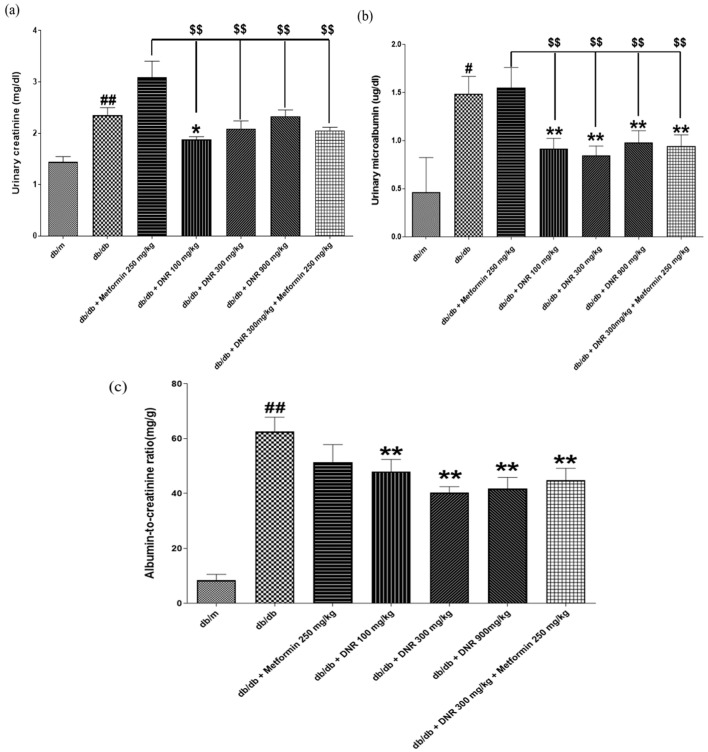
Effects of DNR treatment on urinary and renal biomarkers in *db*/*db* mice. (**a**) Urinary creatinine levels; (**b**) urinary microalbumin levels; (**c**) albumin-to-creatinine ratio. #: Significant difference compared with the *db*/*m* (Student’s *t*-test), *p* < 0.05. ##: Significant difference compared with the *db*/*m* (Student’s *t*-test), *p* < 0.01. *: Significant difference compared with the *db*/*db* (one-way ANOVA), *p* < 0.05. **: Significant difference compared with the *db*/*db* (one-way ANOVA), *p* < 0.01. $$: Significant difference compared with the *db*/*db* + Metfomin (one-way ANOVA), *p* < 0.01.

The present study illustrates pronounced renal impairment in *db*/*db* mice, demonstrated by raised urinary creatinine concentrations (Figure 6a), which are indicative of reduced glomerular filtration rate (GFR) often seen in diabetic nephropathy.

Diabetic nephropathy presents as progressive kidney function decline and albuminuria secondary to renal cellular injury. Loss of podocytes, which are critical components of the renal filtration barrier, compromises glomerular filtration and facilitates the onset of albuminuria [13,14].

Administration of DNR at 100 mg/kg resulted in a significant reduction in creatinine concentrations (*p* < 0.05), suggesting renoprotective effects potentially through improved glomerular filtration or attenuation of renal inflammation and fibrosis. Urinary microalbumin was substantially higher in the *db*/*db* group relative to controls (Figure 6b), and DNR intervention substantially lowered these concentrations; in contrast, Metformin did not elicit a significant change. This finding demonstrates the robust renal protective efficacy of DNR.

Furthermore, the urinary microalbumin-to-creatinine ratio (ACR), a key indicator of renal status, was markedly increased in the *db*/*db* group (Figure 6c).

### 2.7. Histological Improvements in Renal Tissue Following Administration of DNR

**Figure 7:**
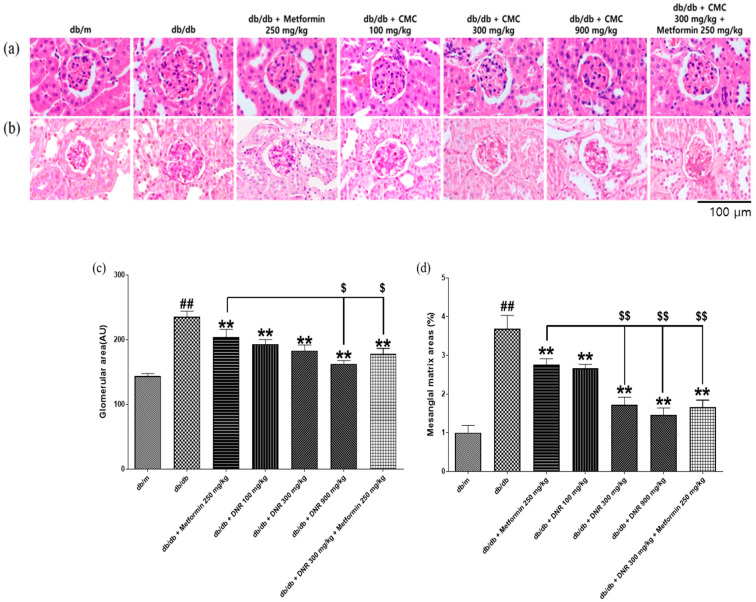
Histological improvements in renal tissue following DNR administration in *db*/*db* mice. (**a**) H&E staining illustrating glomerular lesions; (**b**) PAS staining highlighting extracellular matrix changes; (**c**) quantification of glomerular area; (**d**) assessment of mesangial matrix area percentage. ##: Significant difference compared with the *db*/*m* (Student’s *t*-test), *p* < 0.01. **: Significant difference compared with the *db*/*db* (one-way ANOVA), *p* < 0.01. $: Significant difference compared with the *db*/*db* + Metfomin (one-way ANOVA), *p* < 0.05. $$: Significant difference compared with the *db*/*db* + Metfomin (one-way ANOVA), *p* < 0.01. Scale bar = 100 μm.

Histological evaluation of renal tissues employing Hematoxylin & eosin (H&E) and periodic-acid-Schiff (PAS) staining demonstrated pronounced pathological alterations in the *db*/*db* control group, such as increased glomerular size and diminished capillary territory (Figure 7a,b). These pathological features included glomerular hypertrophy and narrowing of Bowman’s capsule space, accompanied by reduced capillary lumen and higher cellularity owing to mesangial cell proliferation. Treatment with the test substances in all experimental groups led to a statistically significant reduction in glomerular size (*p* < 0.01) and preserved capillary structures within physiological limits. Comparable improvements were also detected in the positive control group (*p* < 0.01). In particular, groups receiving DNR 900 mg/kg and MET 250 + DNR 300 mg/kg demonstrated a significantly greater reduction in glomerular size compared to the MET 250 mg/kg group (Figure 7c, *p* < 0.01). Furthermore, mesangial matrix expansion was markedly ameliorated across all treatment cohorts (*p* < 0.01), with especially pronounced improvements observed in the DNR 300, 900 mg/kg, and MET 250 + DNR 300 mg/kg groups (Figure 7d, *p* < 0.01).

### 2.8. Assessing the Therapeutic Benefits of DNR on Renal Pathology by Immunohistochemical Analysis

**Figure 8:**
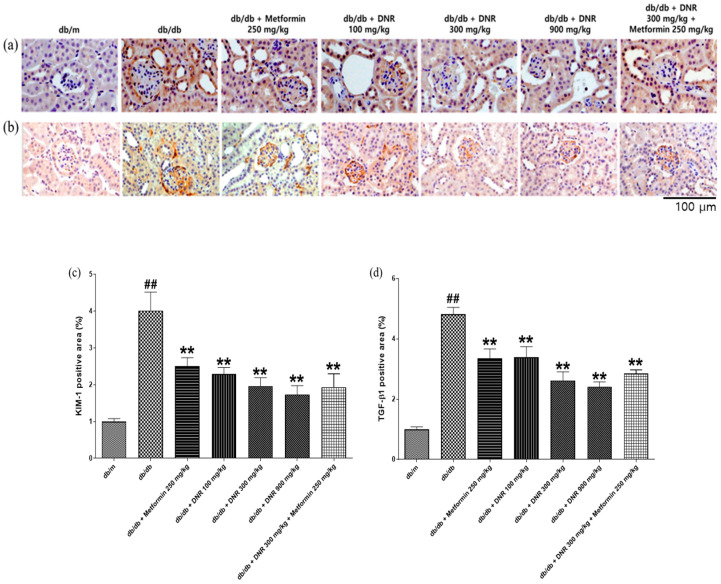
Immunohistochemical analysis of renal pathology in *db*/*db* mice following DNR administration. (**a**) KIM-1 expression; (**b**) TGF-β1 expression; (**c**) quantification of KIM-1 positive area; (**d**) quantification of TGF-β1 positive area. ##: Significant difference compared with the *db*/*m* (Student’s *t*-test), *p* < 0.01. **: Significant difference compared with the *db*/*db* (one-way ANOVA), *p* < 0.01. Scale bar = 100 μm.

Immunohistochemical evaluation of renal tissues (Figure 8) identified notable alterations in the expression patterns of Kidney Injury Molecule-1 (Kim-1) and Transforming Growth Factor-β1 (TGF-β1). The *db*/*db* control group exhibited a significant elevation in areas positive for both Kim-1 and TGF-β1, reflecting exacerbated pathological alterations (Figure 8a,b, *p* < 0.01). In contrast, all treated groups showed a pronounced reduction in these positively stained areas, mirroring the improvement observed in the positive control group (Figure 8c,d, *p* < 0.01). Collectively, these data support a beneficial effect of DNR on renal tissue pathology and underscore its therapeutic potential.

## 3. Discussion

Kidney and glomerular hypertrophy are hallmark features observed in the early stages of diabetes and are regarded as early events that precede glomerulosclerosis and tubulointerstitial lesions characteristic of diabetic nephropathy [15] documented that renal hypertrophy appears prior to hyperfiltration, and there is an association between kidney hypertrophy and increased kidney weight, emphasizing the significance of kidney weight as an indicator of pathological progression in diabetic states. In the present study, the marked decrease in kidney weight detected in the treatment groups suggests that DNR administration may suppress renal hypertrophy or reflect reversal of existing pathological changes, pointing to a potential therapeutic effect. It is important to view alterations in kidney weight not solely as markers of renal function but also as outcomes influenced by shifts in renal cell populations, inflammatory activity, and vascular alterations [16].

The *db*/*db* mouse is a well-established model for diabetic dyslipidemia, in which pronounced elevations in triglyceride and total cholesterol are closely connected to insulin resistance resulting from enhanced hepatic lipid synthesis; these metabolic disturbances are hallmark features of diabetes progression and are associated with liver dysfunction [17]. In our study, DNR administered at 300 and 900 mg/kg produced substantial, dose-dependent decreases in triglyceride and total cholesterol (*p* < 0.01), with the 900 mg/kg dose eliciting the larger effect. These lipid-lowering effects may arise from decreased hepatic lipid accumulation and enhanced expression of low-density lipoprotein receptors, facilitating hepatic cholesterol uptake and influencing systemic lipid homeostasis [18]. Thus, DNR shows promise as a therapeutic candidate for lipid metabolism disorders, although further work is required to define its precise mechanism.

Consistent with its effects on lipids, DNR effectively modulated both serum and urinary glucose concentrations in the *db*/*db* model. The pronounced decrease in serum glucose with high-dose DNR indicates potential utility for hyperglycemia management, a result that parallels activity seen with metformin (MET). The concurrent decline in urinary glucose suggests that DNR may improve glycemic regulation both by lowering systemic glucose and by affecting renal handling of glucose, either increasing reabsorption or reducing urinary elimination. Under normal renal function, glucose is reabsorbed efficiently below a threshold that is frequently exceeded in diabetes, leading to glycosuria; alterations in renal glucose handling have been associated with urinary KIM-1 and the renal glucose threshold in insulin-resistant states. Related studies (e.g., STZ-induced models) report that some treatments can reduce urinary glucose excretion, implying improved renal reabsorption capacity and supporting possible therapeutic benefit [19,20].

Metformin is known to enhance insulin sensitivity and inhibit hepatic gluconeogenesis, but in the context of diabetic kidney disease (DKD), the multifactorial pathophysiology—glomerular injury, inflammation, oxidative stress, and fibrosis—may limit metformin’s impact on markers such as serum creatinine unless dosing or duration are sufficient to affect these pathways [21,22]. In contrast, all metformin- and DNR-treated groups in our study showed marked reductions in BUN compared with *db*/*db* controls, suggesting beneficial effects on renal function. DNR, in particular, produced decreases in serum creatinine and BUN, and combination therapy with metformin further enhanced these outcomes. Collectively, these data support a nephroprotective role for DNR and its potential adjunctive value in diabetes management [23], though longer-term and mechanistic studies are warranted.

In the context of diabetes and diabetic nephropathy, chronic hyperglycemia promotes a cascade of oxidative stress, inflammation, and fibrotic remodeling that collectively drive renal injury. Allicin, the principal sulfur-containing compound identified in DNR, has been extensively reported to counteract these processes by scavenging reactive oxygen species (ROS), enhancing antioxidant enzyme activities such as superoxide dismutase (SOD) and catalase (CAT), and inhibiting TGF-β1/SMAD-mediated fibrosis. Previous studies have shown that allicin alleviates glomerular hypertrophy, mesangial expansion, and extracellular matrix deposition in diabetic kidney models by attenuating oxidative and inflammatory signaling. In this study, although oxidative stress biomarkers were not directly measured, DNR administration led to a marked reduction in renal injury and fibrosis markers (KIM-1 and TGF-β1) and improvement of albumin-to-creatinine ratio, suggesting that DNR mitigates renal damage primarily through anti-fibrotic and renoprotective mechanisms, consistent with the reported biological actions of allicin and related sulfur compounds.

Although this study did not directly measure oxidative stress markers (e.g., SOD, MDA) or inflammatory cytokines (e.g., TNF-α, IL-6), the observed reductions in KIM-1, TGF-β1, and ACR are consistent with the reported antioxidant, anti-inflammatory, and antifibrotic effects of allicin in previous studies, suggesting that these mechanisms may contribute to the renoprotective actions of DNR.

While both DNR and metformin exhibit antioxidant and anti-inflammatory properties, their mechanisms of action are distinct yet complementary. Metformin primarily acts through activation of the AMP-activated protein kinase (AMPK) pathway, which enhances insulin sensitivity, regulates glucose and lipid metabolism, and mitigates metabolic stress. In contrast, DNR exerts its renoprotective effects mainly via the antioxidant and anti-fibrotic activities of its sulfur-containing constituents, particularly allicin, and through chelation of transition metals, thereby reducing metal-catalyzed oxidative reactions. The combination of these two agents, therefore, provides synergistic renal protection by simultaneously targeting metabolic dysfunction and oxidative/fibrotic injury.

The core mechanism underlying the renoprotective effects of DNR involves suppression of oxidative stress and fibrosis-related pathways. DNR markedly reduced the expression of Kidney Injury Molecule-1 (KIM-1) and Transforming Growth Factor-β1 (TGF-β1), indicating attenuation of tubular injury and inhibition of extracellular matrix accumulation. These findings suggest that inhibition of oxidative stress and fibrosis constitutes the principal mechanism for DNR alleviating diabetic nephropathy.

Supporting biochemical findings, DNR treatment produced a notable reduction in albumin-to-creatinine ratio (ACR), whereas metformin did not significantly modify ACR in our experiments. Interestingly, in several parameters, the 300 mg/kg group showed greater improvement than the 900 mg/kg group, indicating a non-linear dose–response pattern. Such non-monotonic relationships are frequently observed in studies on natural product–derived compounds, reflecting the complex interactions among multiple active constituents. This phenomenon may arise from dose-dependent changes in bioavailability, metabolic saturation, or receptor-mediated effects at higher concentrations. Similar U-shaped or hormetic responses have also been reported in natural antioxidant research [24,25,26]. Because ACR is a critical early marker of renal damage and predictor of diabetic renal complications, the reduction observed with DNR underscores its potential to ameliorate microalbuminuria and early functional loss [27,28]. In this study, urinary microalbumin was evaluated as a sensitive and clinically relevant marker of proteinuria, although total urinary protein was not assessed. Histopathologically, diabetic nephropathy manifests as diffuse or nodular glomerulosclerosis, beginning with hypertrophy of glomeruli and tubules driven by mesangial cell activation and augmented synthesis and deposition of extracellular matrix (ECM) proteins. The resulting ECM, visualized by H&E and PAS staining, can progress to Kimmelstiel–Wilson nodules—pathognomonic features of advanced disease—and mesangial expansion reduces capillary density and filtration surface, lowering GFR [29,30]. In our study, the test substances ameliorated pathological changes in renal tissue, suggesting clinical relevance. The chelating action of DNR—binding heavy metals and metabolic wastes and facilitating their removal—may underlie these effects by reducing pathological ECM accumulation characteristic of mesangial expansion and glomerular hypertrophy. By preserving capillary density and filtration surface, DNR could help maintain GFR and stabilize renal architecture [16,31].

At the molecular level, early diabetic nephropathy involves hyperglycemia-driven activation of TGF-β1 signaling with increased expression of ECM components (collagen IV, laminin), producing prominent PAS positivity [32,33]. Kidney Injury Molecule-1 (KIM-1), a sensitive biomarker of proximal tubular injury detectable in urine and plasma, is useful for early detection and monitoring of AKI and CKD [34]. Changes in KIM-1 alongside biochemical and histological improvements would further support DNR’s protective profile; future studies should evaluate KIM-1 and other molecular markers to delineate mechanisms.

HPLC and LC–MS/MS analyses identified allicin as an organosulfur compound present in DNR. Allicin and related sulfur-containing molecules have been reported to scavenge free radicals, inhibit lipid peroxidation, and modulate inflammatory and fibrotic signaling pathways. In line with these reported properties and our histopathological observations, DNR treatment was associated with decreased expression of KIM-1 and TGF-β1, suggesting attenuation of tubular injury and fibrosis. Improvements in urinary albumin, creatinine, and the albumin-to-creatinine ratio further support DNR’s potential to mitigate key pathological features of diabetic nephropathy. Collectively, these findings imply that the renoprotective effects of DNR arise through antioxidant, anti-inflammatory, and anti-fibrotic actions mediated by its sulfur-containing organic compounds, including allicin. This approach highlights allicin as a representative marker compound, while further comprehensive profiling will be necessary to fully elucidate the contribution of multiple sulfur metabolites to the overall efficacy of DNR.

It should be noted that allicin, the principal sulfur-containing compound detected in DNR, is chemically unstable and prone to degradation during extraction and storage. To minimize such degradation, the concentrated extract in this study was immediately freeze-dried and stored at −70 °C, and all analytical samples were freshly prepared before LC–MS/MS analysis. However, the quantitative content of allicin (mg/g extract) was not determined in the present work. Accordingly, allicin is described as a qualitatively confirmed constituent rather than a standardized marker compound. Future studies will aim to quantify allicin and related sulfur compounds using validated analytical methods—such as external-standard calibration—to ensure reproducibility and to assess compositional stability.

Although this study primarily focused on histological evaluation and key fibrosis-related markers such as KIM-1 and TGF-β1, further mechanistic exploration is warranted. Future studies should include the measurement of hydrogen sulfide (H_2_S) levels and TGF-β/SMAD pathway proteins to provide direct molecular evidence for the antifibrotic effects of DNR and to clarify its potential involvement in H_2_S-mediated signaling.

**Figure 9:**
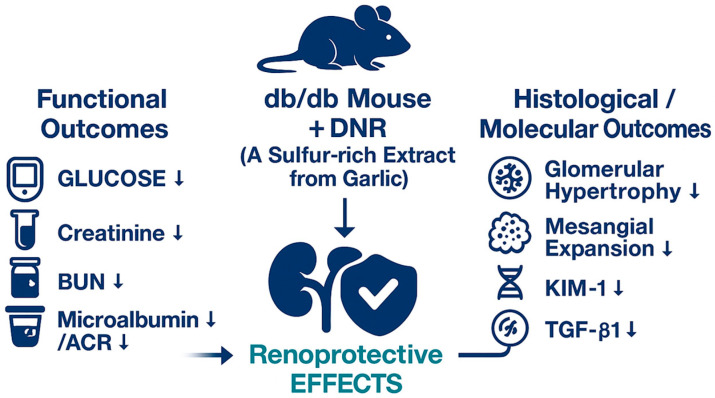
Proposed renoprotective mechanism of DNR (sulfur-rich garlic extract) in *db*/*db* mice. DNR administration improved multiple pathological and functional parameters of diabetic nephropathy. It reduced blood glucose and urinary glucose, decreased serum creatinine and BUN, and alleviated proteinuria (microalbumin and ACR). Histological analysis demonstrated amelioration of glomerular hypertrophy and mesangial expansion, and immunohistochemistry showed downregulation of KIM-1 and TGF-β1. Collectively, these changes indicate that DNR exerts renoprotective effects through metabolic, functional, histological, and molecular improvements.

A schematic summary of these findings is illustrated in Figure 9. DNR, a sulfur-rich extract from garlic, exerted renoprotective effects in db/db mice by improving functional parameters (glucose, creatinine, BUN, and microalbumin/ACR) and alleviating histological alterations, including glomerular hypertrophy and mesangial expansion. Moreover, DNR treatment suppressed renal injury markers such as KIM-1 and TGF-β1, collectively indicating attenuation of renal fibrosis and injury.

In conclusion, our findings indicate that DNR exerts multifaceted beneficial effects in a *db*/*db* diabetic nephropathy model, including reduction in renal hypertrophy (kidney weight), improvement of lipid and glucose profiles, lowering of BUN/creatinine and ACR, and amelioration of histopathological lesions. These effects may be mediated in part by chelation-related reduction in ECM accumulation and modulation of metabolic and inflammatory processes.

While the *db*/*db* mouse is a well-established and widely used model that mimics many metabolic and renal manifestations of human type 2 diabetic nephropathy, certain limitations should be acknowledged. This model primarily reflects obesity-induced hyperglycemia and glomerular injury but does not fully capture the hemodynamic, genetic, and environmental heterogeneity present in human disease. Therefore, the translational relevance of these findings requires cautious interpretation. Future investigations incorporating human renal tissues, patient-derived cell models, and clinical validation will be essential to confirm the applicability of DNR’s renoprotective effects in human diabetic nephropathy.

## 4. Materials and Methods

### 4.1. DNR Preparation

Hong-san garlic, sourced from the Rural Development Administration, was cultivated and harvested in Hongseong for experimental use. The garlic underwent peeling, washing, and crushing processes before being combined with mineral water fortified with organic sulfur and sulfate (SO_4_^2−^), followed by extraction at 40–50 °C. The resulting mixture was filtered utilizing a suction filter (No. 3, Whatman, Maidstone, UK), after which the solvent was removed by vacuum concentration (N-2110, EYELA, Tokyo, Japan) to yield a concentrated extract. This extract was subsequently freeze-dried and powdered with a freeze dryer (FD8518, Ilshinbio-base, Ede, The Netherlands), then preserved at −70 °C in an ultra-low temperature freezer for downstream analyses. The physicochemical characteristics of the mineral water employed for extraction are presented in Table 1.

For dosing, the test material was weighed without applying a purity correction factor. The high-dose formulation (900 mg/kg) was prepared using sterile distilled water, with the medium- and low-dose solutions generated by serial dilution from this stock.

#### HPLC and LC–MS/MS Analysis of Allicin (Methods)

Hongsan garlic extract was diluted with methanol to a final concentration of 1000 μg/mL for analysis. Chromatographic separation was performed on a Shiseido Capcell Pak C18 column (250 × 4.6 mm, 5 μm) under isocratic conditions using 0.1% formic acid in water and acetonitrile (65:35, *v*/*v*) as the mobile phase at a flow rate of 1.0 mL/min. The column oven temperature was maintained at 35 °C, and the injection volume was 10 μL. Detection was carried out at 242 nm with a UV detector.

Mass spectrometric confirmation was conducted under ESI positive ion mode using a Phenomenex C18 column (250 mm × 4.6 mm, 5 μm). The injection volume was 5 μL, the source temperature was 500 °C, and the optimized voltages were: EP –10 V, CE –28.72 V, and CXP –10 V.

### 4.2. Experimental Animals

The *db*/*db* mouse model was utilized to evaluate the therapeutic potential against diabetic nephropathy. 80 male mice of the C57BLKS/J strain were procured from Central Lab. Animal Inc. (Seoul, Republic of Korea). Upon arrival at 7 weeks of age, all animals underwent health screening and a 6-day acclimatization, after which seventy healthy males were selected for experimentation. Administration of the test substances began at approximately 8 weeks of age. The Control group (C57BLKS/J-*m*+/*db*) included non-diabetic mice with unaltered metabolic activity, serving as the comparator. The Negative Control group (C57BLKS/J-*db*/*db*), exhibiting diabetic nephropathy, served as the baseline to assess the efficacy of the test compounds without intervention. Assignment to experimental groups was performed randomly, based on pre-dosing clinical observations.

Mice were maintained under standardized environmental conditions: temperature of 22 ± 3 °C, relative humidity at 50 ± 20%, and 10–15 air exchanges per hour. The light/dark cycle was controlled at 12 h (08:00 to 20:00 light phase). Environmental noise did not exceed 60 dB, and ammonia concentrations remained under 5 ppm. Animals were provided with Teklad Certified Irradiated Global 18% Protein Rodent Diet (Envigo, Indianapolis, IN, USA) and housed with Coarse Sani-Chips Bedding (Envigo Co., Ltd., Indianapolis, IN, USA) to maintain sanitary conditions.

The study comprised seven groups of male animals, each containing 10 individuals identified by distinct animal IDs. Group 1 (G1) functioned as the negative control, consisting of *db*/*m* genotype animals that did not receive any treatment. Group 2 (G2) included *db*/*db* diabetic model animals maintained without treatment. Group 3 (G3) acted as the positive control, in which *db*/*db* animals were administered Metformin at a dose of 250 mg/kg. Groups 4 to 6 (G4–G6) involved *db*/*db* animals receiving DNR at low (100 mg/kg), medium (300 mg/kg), and high (900 mg/kg) doses, respectively. Group 7 (G7) was treated with a combination of Metformin (250 mg/kg) and DNR (300 mg/kg). This experimental setup facilitates direct comparison among untreated diabetic models, positive controls, and different DNR dose levels.

### 4.3. Induction of Diabetic Nephropathy and Substance Administration

The C57BLKs/J mouse strain carries a chromosomal mutation on chromosome 4 that alters the leptin receptor encoded by the *db* gene. This mutation results in persistent hyperinsulinemia, obesity, hyperglycemia, hyperlipidemia, and insulin resistance in the mice. These metabolic disturbances ultimately lead to the development of diabetic nephropathy. The test substance was delivered orally by gavage to ensure dose precision. All administrations were performed at a consistent time daily for 8 weeks, while the control group received 5 mL/kg of sterile distilled water as a vehicle.

### 4.4. Essential Physiological Observations

General observations were conducted once daily after administration for the entire study period. Data collected included mortality, observed symptom types, onset dates, and severity, which were documented individually for each animal. Body weights for all animals were recorded at arrival, during group allocation, on the first administration day, on a weekly basis after dosing, and at necropsy. In addition, caudal artery blood pressure measurements were taken for all subjects before dosing and subsequently once per week.

The doses of metformin (250 mg/kg) and DNR (100, 300, and 900 mg/kg) used in this study fall within the safe and validated range reported in previous *db*/*db* mouse model studies. Throughout the eight weeks of oral administration, no mortality or specific clinical symptoms were observed in any group. In addition, there were no significant changes in body weight, indicating that the doses employed in this experiment are within a safe range without evidence of toxicity.

### 4.5. Clinical Pathology Analysis

Urine samples were obtained from 10 animals per group using metabolic cages for fresh urinalysis. Urine was analyzed using urinalysis test strips (Multistix 10SG, Siemens, Munich and Berlin, Germany) and a urine chemistry analyzer (CliniTek Adrantus, Siemens, Munich and Berlin, Germany) to quantify the following parameters: glucose, creatinine (CRE), and albumin (uALB).

Blood samples were collected from animals designated for necropsy following an overnight fast. On the necropsy day, the animals were anesthetized through isoflurane inhalation, and after verifying anesthesia, blood was drawn from the abdominal aorta. The collected samples were distributed into EDTA-K2 tubes (BD, Microtainer, Franklin Lakes, NJ, USA), 3.2% sodium citrate tubes (Greiner bio-one, Vacuette, Kremsmünster, Austria), and serum separator tubes (Sekisui, Insepack, Roermond, The Netherlands) for subsequent analyses. Serum was prepared by centrifuging the blood in serum-separating tubes at 3000 rpm for 10 min, which was subsequently analyzed with a biochemical analyzer (Hitachi 7180, HITACHI, Tokyo, Japan). Parameters measured included albumin (ALB), blood urea nitrogen (BUN), creatinine (CRE), glucose (GLU), total cholesterol (CHO), and triglyceride (TG).

### 4.6. Histopathological Evaluation

After the administration period concluded, necropsies were conducted on all surviving animals from both the control and test substance-treated groups to examine internal organ findings. The kidneys were isolated, weighed, and relative organ weights were calculated against body weight. Organs and tissues were fixed with 10% neutral buffered formalin to ensure proper preservation.

All tissues underwent sectioning, followed by hematoxylin & eosin and periodic acid-Schiff staining, and were subsequently imaged at 200× magnification. The resulting images were assessed using the Axio Vision SE64 (ZEISS, Oberkochen, Germany) software for measurement and evaluation. Additionally, TGF-β1 and KIM-1 protein expression was quantified to evaluate the extent of cell infiltration.

### 4.7. Statistical Analysis

Statistical evaluations were conducted using SPSS 12.0 K software (SPSS, Chicago, IL, USA), in accordance with experimental guidelines. Student’s *t*-test was applied for group comparisons between control and treatment groups. For analyses involving more than two groups, parametric multiple comparison methods were employed, and statistical significance was established at *p* < 0.05. Additionally, one-way ANOVA was performed to detect group differences and assess variance. Where ANOVA indicated significant results, Duncan’s test was used, while Dunnett’s T3 test was selected in cases of heterogeneity of variance.

## 5. Conclusions

This investigation demonstrates the promising therapeutic value of DNR in improving diabetic nephropathy in the *db*/*db* mouse model. Data indicate that DNR treatment leads to significant enhancements in multiple renal health parameters, including decreases in kidney weight, serum creatinine, and blood urea nitrogen, alongside improved lipid metabolism and glycemic control. These outcomes were most pronounced at elevated doses, implying a dose-dependent response to DNR.

Histopathological analysis reinforces these results, showing marked restoration of renal tissue architecture, including diminished glomerular hypertrophy and reduced mesangial matrix expansion. The immunohistochemical assessment verifies the reduction in principal renal injury markers, such as KIM-1 and TGF-β1, thereby emphasizing DNR’s capacity to protect against renal fibrosis and injury.

As discussed previously, sulfur-containing constituents, especially allicin, are integral to the health-promoting effects ascribed to garlic. These molecules possess antioxidant and anti-inflammatory properties that contribute to renal protection and suppression of fibrotic processes. The present research demonstrated that extracts derived from the high-sulfur ‘Hongsan’ garlic variety conferred nephroprotective effects in a diabetic nephropathy context. Administration of DNR led to enhanced renal outcomes, including attenuation of inflammation and fibrosis, supporting the view that sulfur-rich constituents such as allicin play a substantial role in the management of kidney pathologies.

When compared with standard treatments such as metformin, DNR exhibited equivalent or, in certain respects, superior potential in restraining the progression of diabetic nephropathy. This finding advocates for DNR’s role as a robust natural therapeutic option capable of augmenting current pharmacological strategies.

Cumulatively, these findings highlight the importance of identifying natural interventions like DNR for diabetic kidney disease management. Future research should aim to clarify the underlying mechanisms of action and assess the sustained efficacy and safety of DNR, thereby strengthening its inclusion within clinical strategies for diabetic nephropathy.

## Figures and Tables

**Table 1:** Physicochemical characteristics of mineral water utilized in garlic extraction.

	Al	As	Be	Cd	Co	Cu	Fe	Mn	Pb	Sb	S
mg/L	<0.01	<0.01	<0.01	<0.01	0.03	0.16	ND	0.01	0.14	ND	558
	Se	Sr	Ti	Zn	Ca	Mg	P	SO_4_^2−^	NO_2_^−^	TOC	pH
mg/L	0.3	1.6	1.9	0.46	897	18	0.03	1010	0.3	1.54	7.5

## Data Availability

The data that support the findings of this study are available from the corresponding author upon reasonable request.
